# Point-Of-Care Ultrasound Use for Detection of Multiple Metallic Foreign Body Ingestion in the Pediatric Emergency Department: A Case Report

**DOI:** 10.21980/J83D2D

**Published:** 2023-10-31

**Authors:** Sarah Bella, Joseph Heiney, Amy Patwa

**Affiliations:** * New York Presbyterian Brooklyn Methodist Hospital, Department of Emergency Medicine, Brooklyn, NY; ^ Morristown Medical Center, Department of Emergency Medicine, Morristown, NJ

## Abstract

Foreign body ingestions are common complaints in the pediatric emergency department that can be difficult to diagnose in patients with vague symptoms. Magnetic foreign body ingestion can cause significant morbidity and mortality in children. Point-of-care ultrasound (POCUS) is an imaging modality that can be used to readily diagnose emergent abdominal pathology in a timely matter at the patient’s bedside. In the case described in the report below, a 6-year-old male swallowed multiple round magnets, causing severe abdominal pain. This was detected on POCUS. An abdominal radiograph also confirmed the POCUS findings of multiple hyperechoic round foreign bodies. Key clinical features and sonographic findings are discussed that may help clinicians using POCUS to identify ingested foreign bodies in Pediatric Emergency Departments.

**Topics:**

Point-of-care ultrasound, pediatric emergency medicine, foreign body ingestion.

[Fig f1-jetem-8-4-v1]

**Figure f1-jetem-8-4-v1:**
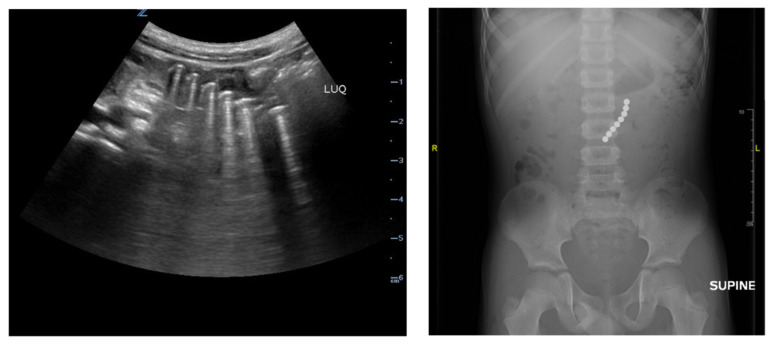


## Brief introduction

Foreign body ingestion is a common complaint in the emergency department, with over 75% of the approximately 100,000 foreign body ingestions reported in the United States each year occurring in children.^1^ Ingestion of multiple foreign bodies is uncommon but usually occurs in children with behavioral problems or developmental delay.^1^ A detailed history and physical exam may not be enough to diagnose foreign body ingestions making radiography necessary for diagnosis^2^. Point-of-care ultrasound is regularly used in the emergency department and, when performed by trained physicians, can help diagnose ingested foreign bodies in pediatric patients^2^, ^3^. Point-of-care ultrasound is 90% sensitive for the detection of foreign bodies^4^. Recent literature has demonstrated multiple benefits of POCUS in suspected foreign body ingestion compared to X-ray. This report focuses on the benefits of using POCUS to diagnose foreign body ingestions in pediatric emergency department patients.

## Presenting concerns and clinical findings

A 6-year-old male with a past medical history of short attention span and behavioral issues presented to the emergency department with a complaint of abdominal pain and vomiting. The patient’s mother at the bedside stated that the patient began complaining of abdominal pain five days prior with decreased appetite. On the day of the presentation, the patient had several episodes of vomiting with continued abdominal pain. Mom noted a decrease in his urination and bowel movements. The mother denied the patient experiencing a history of similar symptoms, sick contacts, known foreign body ingestion, bloody emesis, diarrhea, bloody or pale stools, fever, or respiratory symptoms. The patient’s abdomen was non-focally tender, with voluntary guarding. A bedside POCUS of his abdomen followed by an abdominal radiograph was performed. Significant labs include WBC 12.15 k/mm^^3^ 88% PMNs.

## Significant findings

Bedside POCUS was performed on the patient’s abdomen using the curvilinear probe. The left upper quadrant POCUS image demonstrates multiple hyperechoic spherical objects with shadowing and reverberation artifacts concerning multiple foreign body ingestions. Though the patient and mother initially denied knowledge of foreign body ingestion, on repeated questioning after POCUS findings, the patient admitted to his mother that he ate the spherical magnets he received for his birthday about one week ago. The patient swallowed these over the course of two days. The presence of multiple radiopaque foreign bodies was confirmed with an abdominal X-ray.

## Patient course

After the diagnosis was determined, the patient was treated in the Emergency Department with 20 cc/kg IVF bolus and ondansetron, and a surgical consultation was obtained. General surgery took the patient emergently to the OR for laparotomy, where they found and repaired seven enterotomies and removed eight magnets, some of which were extraluminal. The patient was discharged home several days later. One week later, he re-presented to the ED with vomiting. At that time, he was diagnosed with a bowel obstruction requiring operative intervention for lysis of adhesions.

## Discussion

Foreign-body ingestion is a common complaint in the Pediatric Emergency Department, with multiple foreign-body ingestions occurring in children with behavioral problems or developmental delays. Despite a thorough history and physical exam, foreign-body ingestions may be challenging to diagnose as children may present with non-specific symptoms. Radiography is the initial method of diagnosis for foreign-body ingestion, however, additional lab work and imaging testing may be required. POCUS is a beneficial diagnostic tool to efficiently diagnose foreign body ingestions while limiting costs and radiation exposure in pediatric patients who may have ingested dangerous materials.

A recent study found the sensitivity and specificity of POCUS for foreign-body ingestions to be 90% and 100%, respectively^4^. Although the sensitivity of POCUS for detecting foreign-body ingestion is lower than radiography, POCUS was able to help determine the presence of foreign bodies in this case^4^. The type of material ingested may be determined based on point-of-care ultrasound findings. For instance, radiopaque objects such as metallic foreign bodies will be identified as hyperechoic structures with posterior shadowing and reverberation artifact^5^. Non-radiopaque structures such as glass, plastic, or wood may appear hyperechoic but may not be associated with reverberation artifacts^6^. Retained foreign bodies may also be associated with an inflammatory process noteable on POCUS as a hypoechoic halo of fluid around the foreign body^7^.

Although radiographs and CT scans have been used for foreign body ingestion, recent literature has demonstrated multiple benefits of point-of-care ultrasound in suspected foreign body ingestion. Point-of-care ultrasound is cost-effective and may be repeated numerous times at the bedside for serial exams. Point-of-care ultrasound also provides the benefit of accurate localization of the foreign body and real-time assessment of object movement with peristalsis^8^. POCUS also limits radiation exposure to pediatric patients. The quick diagnosis of foreign body ingestion by point-of-care ultrasound can reduce the time to diagnosis and definitive treatment such as endoscopic removal^9^. Indications for endoscopic or surgical removal of ingested foreign bodies in children depend on the type, shape, number, and position of foreign bodies combined with patient presentation^10^,^11^. Some limitations of POCUS include user dependency and difficulty visualizing smaller superficial foreign bodies requiring a water bath technique to magnify the object and identify a focal zone^12^.

High-powered magnets are common in household appliances and toys, and when one or multiple rare, ingested pose unique diagnostic and management challenges. Ingestion of two or more of these magnets (or one magnet and a metallic object), especially if ingested at different times, carries a high risk of obstruction, volvulus, pressure necrosis, perforation, and hemorrhage if the magnets attract across loops of bowel^10^, ^11^, ^13^. In cases of multiple magnet ingestion (or one magnet with metallic object), the magnets should almost always be preemptively removed^10^, ^11^, ^14^. Urgent endoscopic removal should be attempted if the magnets were ingested together or temporally very close and are all identified in the esophagus or stomach. In cases where the magnets are beyond the stomach, if the patient is asymptomatic, they can undergo serial exams and radiographs to ensure progression^10^, ^11^, ^14^. Endoscopy and colonoscopy can be attempted if magnets are accessible in these patients. Symptomatic patients should undergo urgent surgery for removal^10^. The serious complications associated with foreign body ingestion highlight the importance of applying point-of-care ultrasound early in suspected cases to expedite management and avoid adverse outcomes.

Patients who ingest multiple magnets are at risk for significant bowel injury, bleeding, infection, and sequelae. Even with prompt diagnosis and definitive management, patients such as the young boy, in this case, may develop long-term problems related to the physical damage caused. Increasing awareness among Emergency Physicians to use point-of-care ultrasound to detect foreign body ingestion is of great importance to community health.

## Supplementary Information





## References

[1] Conners G Mohseni M Pediatric Foreign Body Ingestion STAT Pearls 2021 July 33760501

[2] Buonsenso D Chiaretti A Curatola A Morello R Giacalone M Parri N Pediatrician performed point-of-care ultrasound for the detection of ingested foreign bodies: case series and review of the literature J Ultrasound 2021 Mar 24 1 107 114 10.1007/s40477-020-00452-z 32212088 PMC7925727

[3] Horowitz R Cico SJ Bailitz J Point-of-care Ultrasound: A New Tool for the Identification of Gastric Foreign Bodies in Children? J Emerg Med 2016 Jan 50 1 99 103 10.1016/j.jemermed.2015.07.022 26409678

[4] Xin Y Qun Jia L Wei Dong Y Application of high-frequency ultrasound in the diagnosis of gastrointestinal magnet ingestion in children Frontiers in Pediatrics 2023 10 10.3389/fped.2022.988596 PMC9880474 36714638

[5] Harcke HT Rooks VJ Sonographic localization and management of metallic fragments: a report of five cases Mil Med 2012 Aug 177 8 988 92 10.7205/milmed-d-11.00450 22934382

[6] Mohammadi A Ghasemi-Rad M Khodabakhsh M Non-opaque soft tissue foreign body: sonographic findings BMC Med Imaging 2011 Apr 10 11 9 10.1186/1471-2342-11-9 21477360 PMC3079678

[7] Lewis D Jivraj A Atkinson P Jarman R My patient is injured: identifying foreign bodies with ultrasound Ultrasound 2015 Aug 23 3 174 80 10.1177/1742271X15579950. 27433254 PMC4760591

[8] Piotto L Gent R The value of ultrasound in the investigation of ingested foreign bodies in children Sonography 2018 5 2 51 60 10.1002/sono.12139

[9] Le Coz J Orlandini S Titomanlio L Rinaldi VE Point of care ultrasonography in the pediatric emergency department Ital J Pediatr 2018 Jul 27 44 1 87 10.1186/s13052-018-0520-y 30053886 PMC6064059

[10] Hussain SZ Bousvaros A Gilger M Management of ingested magnets in children J Pediatr Gastroenterol Nutr 2012 Sep 55 3 239 42 10.1097/MPG.0b013e3182687be0 22785419

[11] Bolton SM Saker M Bass LM Button battery and magnet ingestions in the pediatric patient Curr Opin Pediatr 2018 Oct 30 5 653 659 10.1097/MOP.0000000000000665 30188872

[12] Shelhoss SC Burgin CM Maximizing Foreign Body Detection by Ultrasound With the Water Bath Technique Coupled With the Focal Zone Advantage. A Technical Report Cureus 2022 Nov 16 14 11 e31577 10.7759/cureus.31577 36540531 PMC9757647

[13] Uyemura MC Foreign body ingestion in children Am Fam Physician 2005 Jul 15 72 2 287 91 16050452

[14] Wyllie R Foreign bodies in the gastrointestinal tract Curr Opin Pediatr 2006 Oct 18 5 563 4 10.1097/01.mop.0000245359.13949.1c 16969173

