# Supplementary figures and images for: Point-Of-Care Ultrasound Use for Detection of Multiple Metallic Foreign Body Ingestion in the Pediatric Emergency Department: A Case Report
Source: J Educ Teach Emerg Med. 2023 Oct 31;8(4):V1–4. doi: 10.21980/J83D2D (PMC10631811; doi:10.21980/J83D2D)

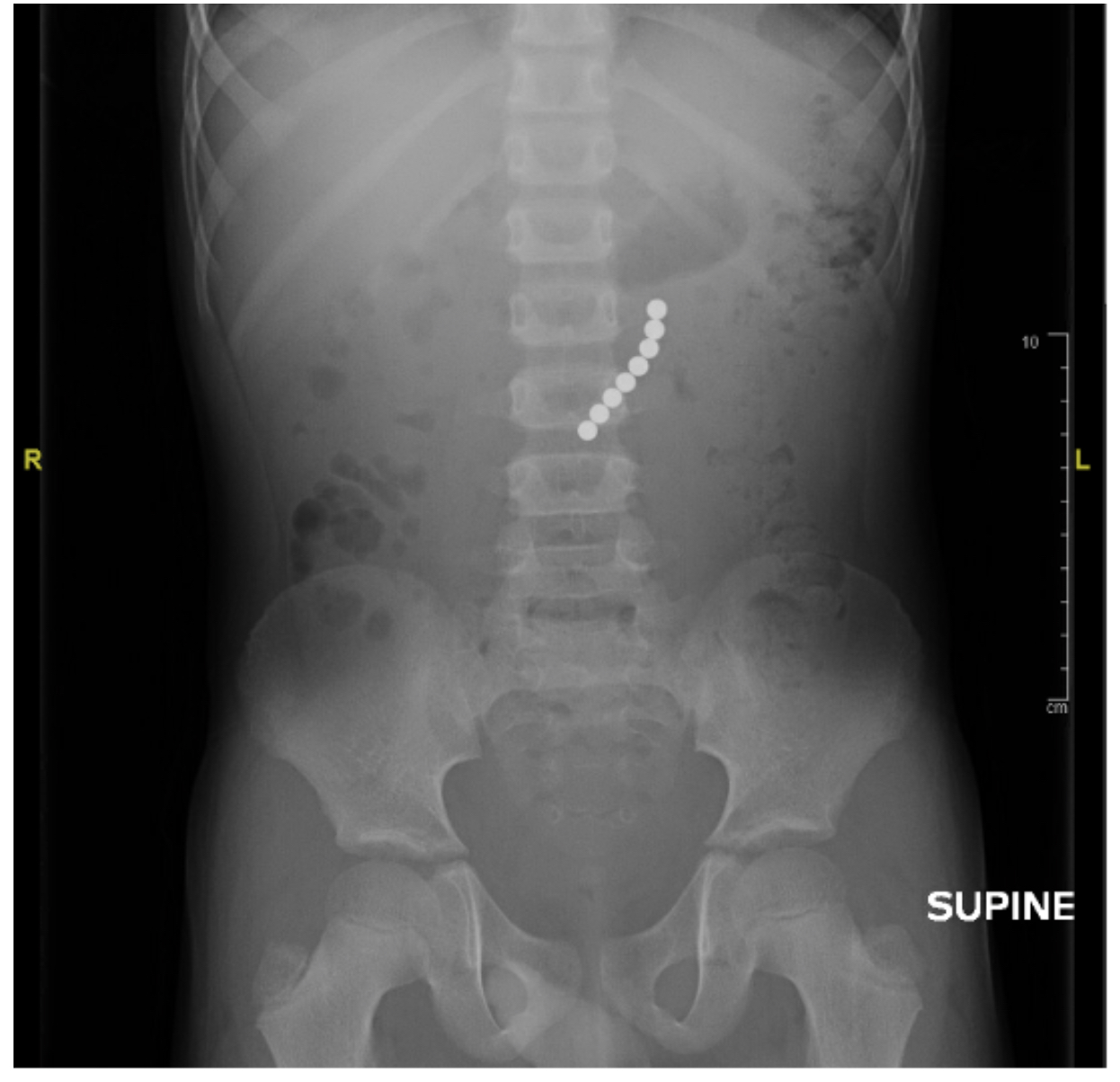

Supplement: Supplementary file 1 [file jetem-8-4-v1-supp1.jpeg]

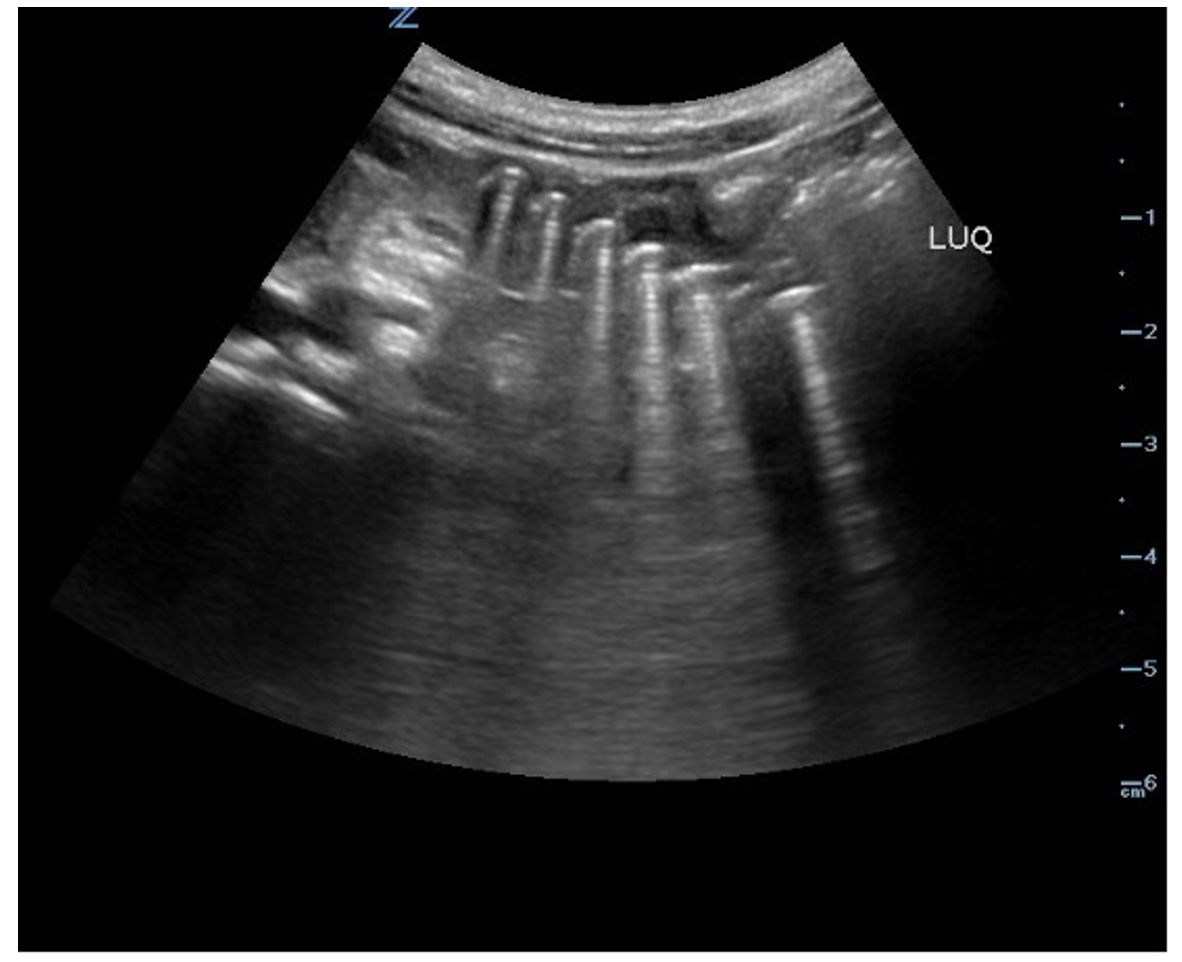

Supplement: Supplementary file 2 [file jetem-8-4-v1-supp2.jpeg]
